# Do Environmental Stringency Policies and Human Development Reduce CO_2_ Emissions? Evidence from G7 and BRICS Economies

**DOI:** 10.3390/ijerph18136727

**Published:** 2021-06-22

**Authors:** Funda Hatice Sezgin, Yilmaz Bayar, Laura Herta, Marius Dan Gavriletea

**Affiliations:** 1 Department of Industrial Engineering, Istanbul University-Cerrahpaşa, 34320 Istanbul, Turkey; 2 Department of Economics, Bandirma Onyedi Eylul University, 10200 Bandirma, Turkey; yilmazbayar@yahoo.com; 3 Department of International Relations and German Studies, Faculty of European Studies, Babeș-Bolyai University, 400084 Cluj-Napoca, Romania; laura.herta@ubbcluj.ro; 4 Department of Business, Faculty of Business, Babeș-Bolyai University, 400084 Cluj-Napoca, Romania; marius.gavriletea@ubbcluj.ro

**Keywords:** environmental stringency policies, human development, CO_2_ emissions, panel cointegration and causality analyses

## Abstract

This study explores the impact of environmental policies and human development on the CO_2_ emissions for the period of 1995–2015 in the Group of Seven and BRICS economies in the long run through panel cointegration and causality tests. The causality analysis revealed a bilateral causality between environmental stringency policies and CO_2_ emissions for Germany, Japan, the United Kingdom, and the United States of America, and a unilateral causality from CO_2_ emissions to the environmental stringency policies for Canada, China, and France. On the other hand, the analysis showed a bilateral causality between human development and CO_2_ emissions for Germany, Japan, the United Kingdom, and the United States of America, and unilateral causality from CO_2_ emissions to human development in Brazil, Canada, China, and France. Furthermore, the cointegration analysis indicated that both environmental stringency policies and human development had a decreasing impact on the CO_2_ emissions.

## 1. Introduction

The environmental problems, together with food security, global health, education, gender equality, poverty, are some of the most pressing global issues in the world.

Global warming, pollution, ozone and natural resources depletion, habitat and biodiversity loss, deforestation, human overpopulation, and waste disposal have been identified as the main global environmental issues [1,2] that need to be solved on a global scale. All countries are facing environmental challenges and need to ensure environmental protection, but there are significant differences from country to country related to strategies adopted to address all these challenges.

Researchers found a major gap between developed and developing countries in the ways in which environmental issues are perceived and understood, and the adoption of concrete strategies and actions for addressing environmental problems [3,4,5]. The problem of environmental degradation and protection must be viewed in a more complex and dynamic way: most developed countries have adopted environmental policies designed to protect the environment, but at the same time these countries had, and still have, the highest carbon dioxide emissions per capita [6]. In these circumstances, differentiated responsibilities and obligations must be imposed for countries based on their level of development.

New patterns of development based on environmental protection must be found, and developed nations must lead in finding proper regulations, standards, policies, technologies that facilitate and encourage the transition to environmentally sustainable economies and societies. Developing and least developed countries must be helped in their transition process to sustainable development, since they are confronted with issues such as the dominance of primary sectors, a significant percent of informal employment [7], a lack of new technologies, and a lack of knowledge that makes the transition more difficult compared to developed countries.

In this context, various environmental policies, including legal and market-based solutions such as property rights, environmental standards, and environmental taxes, have been developed to combat environmental degradation.

Over the last few decades, environmental policies have gained prominence in both government’s strategic agendas and non-state actors’ agendas, with such endeavors as the 2015 Paris Agreement marking ambitious steps in the struggle against greenhouse gas emissions. The substantial effort to reduce such emissions and to limit global warming implies complex and often controversial economic, social and even political stakes and has left a strong imprint on doctrine. The field of international relations has also been impacted by the tensions between leaders that have been opposed to harsher measures associated with environmental protection, or even denied climate change and the science behind it altogether, and those who have developed and implemented strategies, plans, etc. to respond to different requirements set out by national and even supranational regulatory frameworks.

In 2005, the G8 leaders agreed on a set of common goals and principles designed to address climate change, clean energy and sustainable development in Gleneagles, UK. The result was the Gleneagles Plan of Action, according to which, the G8 countries pledged to encourage and support the development of more efficient and lower-emitting vehicles [8]. This goal was consistent with previous commitments and joint efforts of G8, as declared and included in communiqués following summits and high level meetings: undertaking “domestically the steps necessary to reduce significantly greenhouse gas emissions”, as pledged in 1998 in Birmingham; promoting “increasing global participation of developing countries in limiting greenhouse gas emissions”, as decided in 1999 in Cologne; collaborating with international institutions in order “to encourage and facilitate investment in the development and use of sustainable energy”, as agreed upon in 2000 in Okinawa [9].

The European Union (EU) has a long-term commitment to combat climate change, reflected by a range of indicators that have so far been included in two multiannual agendas, i.e., the Lisbon Strategy and Europe 2020, focusing mainly on the reduction in greenhouse gas emissions compared to the year of reference, 1990, energy efficiency and the share of renewable energy in total consumption [10]. The capacity of the EU to accomplish its climate change goals, albeit hindered (or perhaps unexpectedly aided) by a series of crises—from financial crises to the coronavirus outbreak—has been proved by the progress that has been made in the use of two main indicators provided by Europe 2020. In 2019, the EU’s overall greenhouse gas emissions were registered at 24% below 1990 levels, i.e., exceeding the target set for 2020, amid the reduction in emissions generated by the transportation sector during the coronavirus pandemic. Renewable energy accounted for 19.7% of all energy consumed in the EU in 2019, while energy efficiency remains insufficiently tackled [11]. The EU has decided that 30% of all expenditure from the 2021–2027 multiannual financial framework—amounting to 1.08 billion euros—should be spent on climate-related goals, the most consistent financial allocation in this regard so far [12].

The research goal of this study is to evaluate the impact of environmental policies and human development on the CO_2_ emissions for the period of 1995–2015 in a sample of G7 and BRICS economies, the top global CO_2_ emitters. The UNDP Human Development Report released in 1990 stipulated that: “The basic objective of development is to create an enabling environment for people to enjoy long, healthy and creative lives.” [13]. Since the Human Development Index is considered the most used proxy for human development in the related literature [14], we have decided to use this indicator for our analyses.

The human development index is a statistical tool developed by the United Nations in 1990 [13] that assesses countries’ social and economic development based on three key dimensions that have been used in our study to capture the broader view of human development.

We focused our research on this particular sample of countries since developed and major emerging economies are considered the largest contributors to CO_2_ emissions [15]. Over the last few decades, countries such as China, the United States, and India significantly increased their CO_2_ emissions [16] and we find this trend around the world. Not only the level of change CO_2_ in the atmosphere but also the rate at which emissions are changing are the main concerns [15] that force us to adopt and implement urgent and innovative measures to reduce emissions. Ecosystems are capable of adapting to different changes in the environment, but increased emissions from the last few decades does not offer too much time to adapt.

Urgent measures and actions are still needed on a global scale, but also, studies that investigate their impact on CO_2_ emissions are necessary. Since environmental policy stringency and environmental taxes are considered the main policy instruments for fighting against environmental degradation [17] and there is a lack of studies on this issue, we focused our research on the relationship between environmental stringency policies and CO_2_ emissions. Additionally, we considered it appropriate to focus attention on human development based on the fact that usually, countries’ level of development influence the demand for products and services that in turn will influence the CO2 emissions. It could be possible for developed countries to invest more in efficient technologies and products so they can decouple output from greenhouse gas emission, but still, more research is necessary to investigate this trend. In this context, we intend to make a contribution to the existing literature in two ways: firstly, the study will be one of the first studies exploring the interaction among environmental policy, human development, and CO_2_ emissions for G7 and BRICS economies, and secondly, we will use econometric tests with cross-sectional dependence that will lead us to obtain relatively more robust results. In this context, the relevant literature is summarized in the forthcoming section, and then the dataset and method are briefly explained in Section 3. The empirical analysis is conducted in the fourth part of the research, and the paper ends with the Conclusions section.

## 2. Literature Review

The effectiveness of environmental policy on CO_2_ emissions has been explored by a limited number of scholars and the general consensus was that environmental policy stringency index developed by OECD [18] can act as a good proxy of environmental policy. Botta and Kózluk [19] claimed that the Environmental Policy Stringency indicators should be considered “a first tangible effort to measure environmental policy stringency internationally over a relatively long-time horizon”, even though they represent a simplification of the multifaceted and multidimensional approach of environmental policies. However, as explained by Wolde-Rufael and Mulat-Weldemeskel [17], despite the fact that environmental tax and environmental policy stringency have become pivotal policy instruments against environmental degradation, there is still a research gap with respect to their combined effectiveness in mitigating emissions especially for emerging economies.

Using data from 1990 to 2012, Ahmed and Ahmed [20] estimated CO_2,_ emissions in China until 2022 and found that stringent environmental policies can contribute to emissions reduction. Based on a recent analysis of 20 OECD countries, Ahmed [21] determined that environmental regulations promote green innovations in the countries examined. Moreover, stringent environmental policies supplemented by environmentally friendly innovations can act as a catalyst for sustainable development. The study of Wang et al. [22], conducted on a panel of 23 OECD countries during 1990–2015, pointed out that environmental policy stringency has a negative impact on CO_2_, NOx, and SOx emissions, and a weak impact on PM2.5 emissions and PM2.5 exposure. The results can be explained by the fact that we have multiple sources of PM2.5 emissions and environmental policies are difficult to be implemented; also, the process of environmental policy stringency composite index construction does not focus attention on PM2.5 limits or restrictions. The empirical results of this study confirm the role of environmental policy stringency and also noted several shortcomings.

Wolde-Rufael and Mulat-Weldemeskel [23] explored the effect of environmental policy stringency on CO_2_ emissions in Brazil, Russia, India, Indonesia, China, Turkey and South Africa over the 1993–2014 period through panel pooled mean group autoregressive distributive lag estimator and discovered an inverted U-shaped interaction between CO_2_ emissions and environmental policy stringency. Same authors [17] analyzed the effect of environmental policy stringency and environmental tax on CO_2_ emissions in seven emerging countries over the 1994–2015 duration through an augmented mean group estimator and discovered a U-shaped interaction between environmental policy stringency and CO_2_ emissions and unilateral causality from the environmental policy stringency index and total environmental tax to CO_2_ emissions. Furthermore, they found that total environmental tax and energy taxes negatively affected the CO_2_ emissions, but no significant causality was discovered among energy and CO_2_ taxes and CO_2_ emissions.

Many studies emphasize the impact of environmental and energy policy on important economic outcomes, such as innovation, productivity and energy efficiency. Albrizio et al. [24] noted that stringency has exhibited an increasing trend in OECD countries over the last two decades. However, stricter environmental policies do not have a significant effect on aggregate productivity, as they have only short-term impacts. The most technologically advanced industries and firms have met with a small increase in productivity, as they are more likely to adapt, while the productivity of the least productive firms has dropped. The erection of barriers to entry and competition, as well as the preoccupation for the economic effects of environmental policies, vary markedly across the countries analyzed, but this variation is found not to be connected to the stringency of environmental policies. Therefore, in order to yield positive economic and environmental results, stringent environmental policies should be accompanied by as few barriers to entry and competition as possible.

The Porter hypothesis suggests that more stringent environmental policies can foster innovation and productivity [25]. Using a group of OECD countries, Albrizio et al. [26] analyzed the impact of changes in environmental policy stringency on productivity growth at industry and firm level and revealed different results based on the level of countries’ technological development. They pointed out that in the most technologically advanced countries, a more rigid environmental policy leads to a short-term increase in industry level productivity, and also, they suggested that the most productive firms have been confronted with a temporary increase in productivity, whilst the less productive ones experienced a productivity growth decline. The study conducted by de Vries and Withagen [27] focused on the relation between environmental policy stringency and sulfur dioxide abatement, used as a proxy for innovation. Three different models of environmental stringency had been analyzed, and only one of them revealed a positive impact of environmental stringency on innovation. In the field of agriculture, Kara et al. [28] analyzed how federal environmental regulations influence agricultural production in the US, and found that stringent environmental regulations may increase the likelihood of adopting certain conservation practices.

Bieth [29] analyzed the impact of economic growth and human development on CO_2_ emissions in six ASEAN economies over the 2007-208 duration through regression analysis and revealed a significant impact of economic growth and human development on CO_2_ emissions.

The relationship between economic development and CO_2_ emissions has been analyzed in the context of Environmental Kuznets Curve validity, and reached different findings depending on the country and the method employed in the analyses. However, the studies have generally employed real GDP per capita for economic development [30,31,32,33]. Our research attempts to fill the gap that still exists related to the use of more complex and reliable measures of economic development by using the human development index to proxy the economic development.

## 3. Data and Method

In our study, the impact of stringency policies and human development on CO_2_ emissions was analyzed in G7 and BRICS economies over the 1995–2015 period through panel cointegration and causality analyses.

In the econometric model, CO_2_ emissions were represented by CO_2_ emissions in terms of metric tons per capita and environmental policy was represented by the environmental policy stringency index provided by the OECD, ranging from 0 (not stringent) to 6 (highest stringency degree) [18] and the stringency shows the degree of environmental policies putting the price on polluting or environmentally harmful behavior (see Botta and Koźluk [19] for detailed information about index construction.). Lastly, human development was proxied by the human development index of UNCTAD [34]. The index is calculated as a geometric mean of normalized indices of life expectancy, education, and gross national income [34]. The logarithmic forms of the series were used in the analyses and the data sources, and their symbols are displayed in Table 1.

The sample of the study consists of G7 nations (Canada, France, Germany, Italy, Japan, the United Kingdom, and the United States) and BRICS economies (Brazil, China, India, Russian Federation, and South Africa). All the series were annual, and the study period was from 1995–2015, because the environmental policy stringency index ended in 2015. The Stata 14.0, Gauss 10.0 and Eviews 10.0 were employed in the econometric analyses and the dataset summary characteristics are shown in Table 2. The mean of CO, EPS, and HDI series were, respectively, 8.63, 1.55, and 0.78 in the sample, but especially CO_2_ emissions showed considerable variations among the countries. Descriptive statistics for variables included in our study, classified by country, is presented in Appendix A. 

In the econometric part of the research, the tests of cross-sectional dependency and homogeneity were firstly conducted to decide which tests to employ for unit root, cointegration and causality analyses. Then, the Pesaran [36] CIPS unit root test, Westerlund and Edgerton [37] LM bootstrap panel cointegration test and Konya [37] bootstrap panel Granger causality test were conducted considering the existence of cross-sectional dependency and heterogeneity.

The Westerlund and Edgerton [37] LM bootstrap panel cointegration test considers the cross-sectional dependency among the series and yields effective results for small sample sizes and it also allows autocorrelation and heteroscedasticity in cointegrating equation. On the other hand, the Konya [38] bootstrap panel Granger causality test takes notice of both cross-sectional dependency and heterogeneity. The test is relied on Seemingly Unrelated Regressions (SUR) estimation, which yields more efficient results in the case of cross-sectional dependency among the series. The causality direction is investigated by Wald tests with bootstrap critical values. Furthermore, the test does not dictate any pretests [38].

## 4. Empirical Analysis

In the applied part of the research, first the cross-sectional dependence among the series was checked by employing tests of Pesaran et al. [39] $LM_{adj.}$, the Pesaran [40] LM CD, and the Breusch and Pagan [41] LM, and the findings are displayed in Table 3. The null hypothesis of cross-sectional independency was declined at 1% significance level and in turn, cross-sectional dependency among the series was reached.

The homogeneity of the cointegration coefficients was checked through delta tilde tests of Pesaran and Yamagata [42], and the findings are displayed in Table 4. The null hypothesis of homogeneity was declined at 1% significance level and the cointegration coefficients were found to be heterogeneous. 

The unit root existence at the series were analyzed by the Pesaran [36] CIPS unit root test, and the findings are displayed in Table 5, and three series were found to be I(1).

The cointegrating relationship among environmental stringency policies, human development and CO_2_ emissions were examined through the Westerlund and Edgerton [37] LM bootstrap cointegration test, and the findings are displayed in Table 6. Furthermore, the critical values were provided with 10,000 simulations and lag and lead values were taken as 2. Asymptotic probability values were derived from standard normal distribution. Therefore, bootstrap P values are considered in the case of cross-sectional existence. The null hypothesis suggesting the existence of significant cointegration relationship among the series were accepted, because the bootstrap *p* value was found to be higher than 10%.

The cointegrating coefficients were estimated through FMOLS (Fully Modified Ordinary Least Squares), and the findings are reported in Table 7. The results revealed that both EPS and HDI had a significant decreasing effect on the CO_2_ emissions, but the decreasing impact of HDI on the CO_2_ emissions was relatively higher when compared with the impact of EPS. The EPS and HDI had the largest decreasing impact on the CO_2_ emissions in the United States of America, but EPS and HDI, relatively, had the least decreasing impact on the CO_2_ emissions in India and China.

The causality among environmental stringency policies, human development, and CO_2_ emissions was checked through the Kónya [38] bootstrap panel Granger causality test given the presence of heterogeneity and cross-sectional dependency, and the findings are reported in Table 8 and Table 9. First, the causality between CO_2_ emissions (CO) and environmental stringency policies (EPS) was checked, and the findings are reported in Table 8. The findings revealed a bilateral causality between CO and EPS for Germany, Japan, the United Kingdom, and the United States of America and a unilateral causality from CO to the EPS for Canada, China, and France.

Then, the causality between human development (HDI), and CO_2_ emissions (CO) was checked through the Kónya [38] bootstrap panel Granger causality test, and the findings in Table 9 revealed a bilateral causality between CO and HDI in countries such as Germany, Japan, the United Kingdom, and the United States of America and a unilateral causality from CO to the HDI for Brazil, Canada, China, and France.

## 5. Conclusions

The 2015 Paris Agreement represented a milestone for the struggle against greenhouse gas emissions. According to the G8 countries’ joint declarations, efforts are consistently taken in order to significantly reduce greenhouse gas emissions and to support sustainable energy and human development. However, as most scholars indicate, there is still a gap between political decisions, on the one hand, and the position of different researchers and analysts regarding the need to further and intensify environmental stringency policies, on the other hand.

The study focuses on the following research questions: do environmental stringency policies and human development reduce CO_2_ emissions? What does causality analysis indicate about the G7 and BRICS economies, namely Canada, France, Germany, Italy, Japan, the United Kingdom, and the United States of America, Brazil, China, India, Russian Federation, and South Africa?

In order to tackle these interrogations, the cointegrating relationship among environmental stringency policies, human development and CO_2_ emissions were examined through the Westerlund and Edgerton [37] LM bootstrap cointegration test and the Kónya [38] bootstrap panel Granger causality test given the presence of heterogeneity and cross-sectional dependency. The causality analysis disclosed a bilateral causality between environmental stringency policies and CO_2_ emissions for Germany, Japan, the United Kingdom, and the United States of America and a unilateral causality from CO_2_ emissions to the environmental stringency policies for Canada, China, and France. Therefore, the causality analysis revealed that environmental stringency policy was found to be effective, especially in the developed countries, in the short run. Moreover, a bilateral causality between human development and CO_2_ emissions was discovered for Germany, Japan, the United Kingdom, and the United States of America, and unilateral causality from CO_2_ emissions to human development was discovered for Brazil, Canada, China, and France.

As we already mentioned in the previous sections, the studies focused on the causal link between CO_2_ emissions and HDI have not reached a clear consensus regarding the causality between two variables, and this can be resulted from different variables, methods, time periods or countries with different characteristics. However, the feedback link between CO_2_ emissions and human development indicates a mutual interaction between two variables, especially in the leading developing countries in the short run, but a unilateral causality from CO_2_ emissions to human development indicates that CO_2_ emissions have significant effect on human development.

Furthermore, the cointegration analysis revealed that both environmental stringency policies and human development were effective in decreasing CO_2_ emissions in the long run. However, both human development and environmental stringency policies were more effective in decreasing the CO_2_ emissions, especially in the developed economies such as the United States of America, Canada, Japan, and the United Kingdom. Therefore, we conclude that the effect of human development and environmental stringency policies on the CO_2_ emissions raise in parallel with development level.

Our findings indicated that environmental stringency policies and human development are important for environmental sustainability. However, environmental stringency policies can negatively affect economic growth and employment through raising the costs at the beginning. However, the countries offset the negative economic effects of environmental stringency policies through innovation, considering the Porter hypothesis and empirical findings over time. On the other hand, improvements in human development also are effective for environment sustainability.

Consequently, there are no uniform environment policies with which the countries can achieve their environment targets. Therefore, countries should design an environmental policy mix considering their country-specific characteristics. Future studies should explore the environmental policies at country level by conducting comparative research in different categories of countries (developed, emerging, least developed, OECD, EU countries, etc.).

## Figures and Tables

**Table 1:** Data description.

Variables	Description	Definition	Data Sources
CO	CO_2_ emissions (metric tons per capita)	“The total amount of carbon dioxide emitted by the country as a consequence of all relevant human (production and consumption) activities, divided by the population of the country” [6].	World Bank [6]
EPS	Environmental policy stringency index	“A country-specific and internationally-comparable measure of the stringency of environmental policy. Stringency is defined as the degree to which environmental policies put an explicit or implicit price on polluting or environmentally harmful behaviour” [18].	OECD [18]
HDI	Human development index	A statistical tool developed by the United Nations in 1990 that assesses countries’ social and economic development based on three key dimensions: a long and healthy life, access to education, and a decent standard of living. Life expectancy index, education index and gross national income (GNI) are taken into consideration to calculate HDI [13].	UNCTAD [35]

Source: own processing.

**Table 2:** Summary statistics of the dataset.

Characteristic	CO	EPS	HDI
Mean	8.634120	1.554643	0.788385
Median	8.768835	1.300000	0.851000
Maximum	20.17875	3.850000	0.938000
Minimum	0.841937	0.330000	0.461000
Std. Dev.	5.031014	1.042403	0.125613
Skewness	0.460112	0.629946	−0.813656
Kurtosis	2.678022	2.073179	2.446227

Source: own processing.

**Table 3:** Results of cross-sectional dependency tests.

Test	Test Statistic	Probability Value
LM _adj_	36.902	0.000
LM CD	34.771	0.000
LM	45.786	0.005

Note: H0: There is cross-sectional independency; H1: there is cross-sectional dependence. Source: own processing.

**Table 4:** Results of homogeneity tests.

Test	Test Statistic	Probability Value
Delta tilde	27.413	0.000
Adjusted delta tilde	29.502	0.000

Note: H0: Slope coefficients are homogeneous; H1: slope coefficients are heterogeneous. Source: own processing.

**Table 5:** Pesaran (2007) CIPS unit root test.

Variables	Level		First differences	
	Constant	Constant + Trend	Constant	Constant + Trend
CO	−1.173	−1.215	−7.662 *	−8.035 *
EPS	−1.564	−1.739	−9.716 *	−9.994 *
HDI	−1.209	−1.296	−8.270 *	−8.619 *

Source: own processing. Note: * it is significant at 1% significance level.

**Table 6:** Pesaran (2007) CIPS unit root test.

$LM_{N}^{+}$	**Constant**			**Constant and Trend**		
	**Test Statistic**	**Asymptotic *p* Value**	**Bootstrap *p* Value**	**Test Statistic**	**Asymptotic *p* Value**	**Bootstrap *p* Value**
	8.361	0.344	0.398	9.557	0.369	0.412

Source: own processing.

**Table 7:** Cointegration coefficients.

Countries	LnEPS	LnHDI
Brazil	−0.073 *	−0.147 *
Canada	−0.113 *	−0.165 *
China	−0.075 *	−0.107 *
France	−0.094 *	−0.135 *
Germany	−0.116 *	0.128 *
India	−0.054 *	−0.113 *
Italy	−0.103 *	−0.124 *
Japan	−0.119 *	−0.169 *
Russia	−0.101 *	−0.120 *
South Africa	−0.106 *	−0.119 *
United Kingdom	−0.121 *	−0.171 *
United States of America	−0.123 *	−0.175
Panel	−0.103 *	−0.142 *

Source: own processing. Note: * it is significant at 5% significance level.

**Table 8:** Bootstrap Granger causality test between lnCO and lnEPS.

Countries	lnCO Does Not Granger Cause lnEPS				lnEPS Does Not Granger Cause lnCO			
	Wald Statistics	Bootstrap Critical Value			Wald Statistics	Bootstrap Critical Values		
		10%	5%	1%		10%	5%	1%
Brazil	37.45	55.24	58.11	61.70	27.45	44.67	46.02	49.36
Canada	78.13 ***	48.14	53.89	55.04	36.19	51.22	54.78	56.09
China	66.59 ***	54.19	58.21	60.88	31.27	48.44	50.19	51.38
France	64.23 ***	46.33	48.47	49.05	40.32	45.73	46.88	48.56
Germany	73.56 ***	50.13	54.66	57.29	69.44 **	68.15	71.36	73.07
India	31.86	48.16	51.45	54.14	39.86	53.86	54.21	56.99
Italy	43.58	52.41	56.78	59.39	37.07	45.38	46.22	47.03
Japan	79.21 ***	55.37	58.19	60.77	75.15 **	73.49	74.99	76.17
Russia	32.17	46.89	47.22	49.25	29.56	40.75	41.58	43.56
South Africa	44.12	61.23	64.89	65.57	31.47	44.86	45.73	48.19
United Kingdom	68.33 **	65.34	69.14	70.88	69.26 **	65.37	68.43	70.83
United States of America	73.89 ***	64.37	66.31	68.11	63.87 **	64.48	66.39	67.85

Source: own processing. Note: *** and ** respectively, indicate that it is significant at 1%, 5%, and 10% significance levels.

**Table 9:** Bootstrap Granger causality test between lnCO and lnHDI.

Countries	lnCO Does Not Granger Cause lnHDI				lnHDI Does Not Granger Cause lnCO			
	Wald Statistics	Bootstrap Critical Value (%)			Wald Statistics	Bootstrap Critical Value		
		10%	5%	1%		10%	5%	1%
Brazil	93.67 ***	67.89	71.45	78.23	46.34	56.78	61.13	64.87
Canada	92.49 ***	56.21	63.55	70.88	24.16	66.28	73.11	75.9
China	79.31 ***	60.47	66.23	69.16	19.85	34.27	40.19	42.52
France	55.73 *	47.24	56.89	60.32	36.82	40.25	38.48	41.19
Germany	73.56 *	68.36	71.44	75.98	61.14 ***	39.26	42.53	45.01
India	22.79	42.79	47.21	49.05	31.88	40.17	44.68	46.17
Italy	62.35 **	54.99	63.67	66.24	33.64	51.59	67.94	69.22
Japan	75.21 ***	46.91	49.16	53.48	77.03 ***	59.68	62.6	64.47
Russia	29.18	48.25	51.18	55.09	36.42	70.14	77.46	79.07
South Africa	34.59	54.64	58.02	61.18	25.18	43.53	48.13	49.44
United Kingdom	73.18 ***	49.23	54.43	58.73	64.85 ***	54.96	53.07	59.21
United States of America	69.15 ***	46.18	49.36	52.77	60.92 **	60.89	62.68	65.15

Source: own processing. Note: ***, **, *, respectively, indicate that it is significant at 1%, 5%, and 10% significance levels.

## Data Availability

Not applicable.

## Appendix A

**Table A1:** Descriptive statistics for analyzed countries.

Country	Characteristic	CO	EPS	HDI
Brazil	Mean	1.9963	0.4414	0.7042
	Median	1.876441	0.420000	0.700000
	Maximum	2.631290	0.630000	0.756000
	Minimum	1.594542	0.380000	0.651000
	Std. Dev.	0.281403	0.069013	0.031227
	Skewness	0.983730	1.594038	0.121792
	Kurtosis	2.827510	4.354830	2.148359
Canada	Mean	16.42349	2.138500	0.889150
	Median	16.67965	1.875000	0.895000
	Maximum	17.56134	3.850000	0.921000
	Minimum	14.79888	0.460000	0.861000
	Std. Dev.	0.951222	1.260419	0.019329
	Skewness	−0.402700	−0.014637	−0.071062
	Kurtosis	1.672255	1.275895	1.775377
China	Mean	4.834477	1.019500	0.647200
	Median	4.751746	0.830000	0.646500
	Maximum	7.557211	2.160000	0.739000
	Minimum	2.648649	0.520000	0.554000
	Std. Dev.	1.926844	0.582955	0.060970
	Skewness	0.228806	1.064472	−0.003893
	Kurtosis	1.494350	2.611028	1.623914
France	Mean	5.679318	2.480500	0.866250
	Median	5.884539	2.785000	0.869000
	Maximum	6.280954	3.700000	0.895000
	Minimum	4.550000	1.150000	0.837000
	Std. Dev.	0.548513	1.001790	0.018038
	Skewness	−0.834628	−0.113231	0.014689
	Kurtosis	2.454713	1.301456	1.724320
Germany	Mean	9.714977	2.621500	0.903850
	Median	9.780996	2.670000	0.913000
	Maximum	10.86023	3.140000	0.938000
	Minimum	8.797642	1.850000	0.846000
	Std. Dev.	0.590215	0.473834	0.030465
	Skewness	0.102840	−0.443551	−0.589654
	Kurtosis	2.052530	1.613958	1.990459
India	Mean	1.213358	0.832500	0.541700
	Median	1.091958	0.630000	0.541000
	Maximum	1.784334	1.820000	0.624000
	Minimum	0.898163	0.460000	0.468000
	Std. Dev.	0.295116	0.398641	0.049592
	Skewness	0.613705	0.938410	0.106093
	Kurtosis	1.937756	2.736987	1.739943
Italy	Mean	7.256863	2.198000	0.859600
	Median	7.668207	2.280000	0.867500
	Maximum	8.216487	3.280000	0.883000
	Minimum	5.140000	1.350000	0.814000
	Std. Dev.	0.981292	0.719778	0.022892
	Skewness	−1.025494	0.115076	−0.654713
	Kurtosis	2.748522	1.389011	2.044067
Japan	Mean	9.485325	2.001500	0.875600
	Median	9.547599	1.680000	0.877000
	Maximum	9.880903	3.500000	0.908000
	Minimum	8.632100	1.330000	0.847000
	Std. Dev.	0.289335	0.711701	0.019422
	Skewness	−1.305026	1.032466	0.104473
	Kurtosis	4.822582	2.437024	1.877646
Russian Federation	Mean	11.31108	0.616000	0.755750
	Median	11.18706	0.600000	0.756500
	Maximum	12.62027	0.920000	0.809000
	Minimum	10.12729	0.330000	0.703000
	Std. Dev.	0.723075	0.134962	0.036007
	Skewness	0.084680	0.553416	−0.046385
	Kurtosis	1.894299	3.630978	1.711029
South Africa	Mean	8.877895	0.695000	0.646700
	Median	8.751202	0.500000	0.643000
	Maximum	9.979458	1.750000	0.701000
	Minimum	7.727642	0.400000	0.611000
	Std. Dev.	0.575199	0.430037	0.026492
	Skewness	0.276880	1.776961	0.604032
	Kurtosis	2.732339	4.541542	2.290577
United Kingdom	Mean	8.332668	2.141500	0.891950
	Median	8.901417	2.090000	0.896500
	Maximum	9.480231	3.830000	0.925000
	Minimum	6.220240	0.810000	0.851000
	Std. Dev.	1.007337	1.146906	0.021982
	Skewness	−0.821794	0.217461	−0.295253
	Kurtosis	2.259445	1.569621	2.108925
United States of America	Mean	18.49938	1.896500	0.902600
	Median	19.35696	1.715000	0.901500
	Maximum	20.17875	3.170000	0.921000
	Minimum	15.98987	1.050000	0.884000
	Std. Dev.	1.467676	0.764339	0.013520
	Skewness	−0.621797	0.194185	0.080692
	Kurtosis	1.684749	1.329994	1.428619

Source: own processing.
